# Analysis of the characteristics of parents’ reasons for refusing HPV vaccination for their daughters: a cross-sectional study

**DOI:** 10.1186/s12889-026-26275-x

**Published:** 2026-01-30

**Authors:** Yihan Hu, Xianglong Li, Lei Zhang, Li Du, Longmei Jin, Ping Zhou

**Affiliations:** 1 School of Public Health, Fudan University, Shanghai, China; 2 Minhang District Maternal and Child Health Hospital, Shanghai, China; 3 Shanghai Center for Women and Children’s Health, Shanghai, China; 4 School of Public Health, National Health Commission Key Laboratory of Health Technology Assessment, Fudan University, Shanghai, China; 5 Shanghai Research Center for Governance of Emerging Technologies in Medicine and Public Health, Shanghai, China

**Keywords:** HPV vaccine, Vaccination intention, Parents, Vaccine hesitancy, Cervical cancer

## Abstract

**Background:**

Parental decision-making is pivotal to the uptake of the human papillomavirus (HPV) vaccine among girls aged 9–14 years. However, the characteristics that distinguish parents who are willing versus unwilling to vaccinate—particularly those who are unwilling—remain insufficiently characterized. This study aimed to examine the underlying reasons and attributes associated with parental willingness and unwillingness, thereby providing evidence on the classification and heterogeneity of HPV vaccine hesitancy.

**Methods:**

In May 2024, we conducted a cross-sectional questionnaire survey using convenience sampling among 1,331 parents of girls aged 9–14 years from seven schools in Shanghai, China. The survey collected sociodemographic characteristics, willingness to vaccinate daughters against HPV and the underlying reasons, and parental knowledge literacy regarding cervical cancer, HPV, HPV vaccination, and cervical cancer screening. Two-step cluster analysis was used to classify parents based on their stated reasons for willingness or unwillingness to vaccinate. We then examined associations between parental characteristics and cluster membership.

**Results:**

Among 1,076 parents of unvaccinated girls aged 9–14 years, 18.12% reported unwillingness to vaccinate. Cluster analysis identified three distinct refusal clusters, labeled as the Moderate and Hesitant type (low knowledge literacy and limited understanding of HPV-related risks), the Trust-Critical type (high knowledge literacy but low institutional trust), and the Social-Norm Sensitive type (high educational attainment and susceptibility to peer and societal influences). Significant between-cluster differences were observed in educational attainment, number of children, and knowledge literacy, indicating that vaccine refusal is structurally diverse rather than uniform.

**Conclusions:**

HPV vaccine refusal among Chinese parents is heterogeneous and appears to be shaped by cognitive, emotional, and social factors. Tailored interventions are therefore warranted. Segmented strategies that address cluster-specific drivers may reduce vaccine hesitancy more effectively than uniform public health messaging.

## Introduction

Human papillomavirus (HPV) is a prevalent sexually transmitted infection that affects nearly all sexually active individuals, often without presenting symptoms [1]. More than 40 HPV types can infect the genital region as well as other areas, including the oral cavity and throat [2]. The International Agency for Research on Cancer has identified 13 HPV types that are associated with cervical and other cancers, such as those of the vulva, penis, anus, and oropharynx [3].

The incidence of cervical cancer peaks among women aged 50–54 years, with the highest mortality observed among those aged 75 years and older [2]. The five-year survival rate for cervical cancer patients between 2014 and 2020 was 67.4% [4]. HPV vaccination remains the most effective method for preventing HPV infection and related malignancies. The World Health Organization (WHO), in its most recent position paper on HPV vaccines (updated in 2022), recommends vaccinating girls aged 9–14 years prior to the onset of sexual activity. Vaccinating preadolescent girls has been shown to be cost-effective, particularly in resource-limited settings [5]. Early vaccination at age 9 enhances infection prevention and improves vaccine series completion rates [6]. In 2020, the WHO introduced a global strategy to eliminate cervical cancer as a public health concern, outlining the 90–70–90 targets to be achieved by 2030. The first target aims for 90% of girls to be fully vaccinated with the HPV vaccine by age 15 [7].

As part of this strategy, the WHO recommends incorporating the HPV vaccine into national immunization programs (NIPs) worldwide. As of 2022, 64% of countries had included the HPV vaccine for girls in their NIPs, and 24% had extended it to boys. In response, the Chinese government launched the “Action Plan for Accelerating the Elimination of Cervical Cancer (2023–2030)” to promote HPV vaccination [8]. Although the HPV vaccine has not yet been included in China’s NIPs, the National Health Commission has taken steps to improve vaccine accessibility [9]. In Shanghai, four types of HPV vaccines are currently available. Vaccination rates are highest among individuals aged 20–24 years, while the rate among those aged 9–14 years remains below 1% [10].

Public awareness and willingness to vaccinate are critical for increasing HPV vaccine uptake. A national cross-sectional study in China examining healthcare workers’ willingness to receive the SARS-CoV-2 vaccine revealed that public confidence in vaccines is crucial for both willingness and actual uptake [11]. Additionally, a survey on hepatitis B vaccination behavior and willingness among rural migrant workers in six Chinese provinces and Beijing demonstrated that improving awareness and knowledge of hepatitis B can effectively enhances vaccination behavior and intention [12].

Health literacy also significantly influences vaccination rates. It is defined as “the degree to which individuals have the capacity to obtain, process, and understand basic health information and services needed to make appropriate health decisions” [13]. Health literacy is essential for accessing healthcare, interacting with professionals, advocating for health, and participating in decision-making [14]. Though often overlooked, it is a critical determinant of preventive behaviors, such as cancer screening [15]. A recent study on health literacy and COVID-19 vaccination found lower vaccination rates among individuals with “insufficient” health literacy [16]. However, international research on the relationship between parental health literacy and HPV vaccination among daughters aged 9–14 years remains limited. This study assesses parents’ awareness of HPV-related knowledge through surveys, which cannot be fully classified as health literacy under its formal definition. Instead, we define it as “knowledge literacy”—a component of overall health literacy. Previous research has identified a positive correlation between health literacy and HPV knowledge, supporting this conceptual distinction [17].

Parental attitudes, particularly in China, play a pivotal role in vaccination decisions for children [18]. It is therefore essential to understand the dynamics of parental decision-making and to design interventions accordingly [19]. A recent meta-analysis found that HPV awareness among Chinese parents is relatively low, with only 45% aware of HPV and 41% aware of the HPV vaccine, although 61% expressed willingness to vaccinate their children [18].

What characteristics do parents who are willing or unwilling to vaccinate their daughters aged 9–14 years against HPV have—particularly those who are unwilling? Can these individuals be clearly differentiated? Is vaccine refusal a homogeneous phenomenon? While existing studies have largely identified key determinants of vaccine refusal [20], the classification of underlying reasons and characteristics remains insufficiently explored. This study contributes to the literature by offering new insights and reference points for understanding the heterogeneity within these populations.

## Methods

### Study design and population

A cross-sectional survey was conducted among parents of girls who had not yet received the HPV vaccine, recruited from seven primary and secondary schools in Shanghai in May 2024. Data collection was completed on May 26, 2024, using the Questionnaire Star online platform. A total of 1,331 questionnaires were collected. After excluding responses with completion times of less than 4 min or more than 20 min (*n* = 136), 1,195 valid questionnaires remained, resulting in an overall valid response rate of 89.78%. Among these, 119 parents indicated that their daughters had already received or scheduled the HPV vaccine; these participants were excluded from the primary analysis. This exclusion was based on the response to the following item: “Has your daughter already received or scheduled the HPV vaccine?” The final analytical sample comprised 1,076 parents whose daughters had not yet received the vaccine. Demographic characteristics of the study participants are presented in Table 1.

**Table 1:** Chi-square tests of participant characteristics and clustering results

	Unwilling (*N* = 195)						Willing (*N* = 881)				
	Moderate and Hesitant type	Social-Norm Sensitive type	Trust-Critical type	χ²	*p*-value	Sum	Highly Affirmative type	Moderately Affirmative type	χ²	*p*-value	Sum
Gender:				2.151	0.401				0.373	0.542	
Male	8 (9.52%)	2 (3.28%)	4 (8.00%)			14	49 (9.21%)	28 (8.02%)			77
Female	76 (90.48%)	59 (96.72%)	46 (92.00%)			181	483 (90.79%)	321 (91.98%)			804
Age:				0.545	0.762				0.009	0.923	
<40	41 (48.81%)	26 (42.62%)	23 (46.00%)			90	299 (56.20%)	195 (54.32%)			494
≥ 40	43 (51.19%)	35 (57.38%)	27 (54.00%)			105	233 (43.80%)	154 (44.13%)			387
Nation:				0.631	0.888				3.113	0.078	
The Han nationality	80 (95.24%)	59 (96.72%)	49 (98.00%)			188	518 (97.37%)	332 (95.13%)			850
Other nationalities	4 (4.76%)	2 (3.28%)	1 (2.00%)			7	14 (2.63%)	17 (4.87%)			31
Marriage:				4.246	0.120				0.704	0.401	
Nonexistent marriage	21 (25.00%)	7 (11.48%)	9 (18.00%)			37	86 (16.17%)	64 (18.34%)			150
In marriage	63 (75.00%)	54 (88.52%)	41 (82.00%)			158	446 (83.83%)	285 (81.66%)			731
Highest educational attainment:				14.710	0.023				7.030	0.071	
Junior high school or below	26 (30.95%)	6 (9.84%)	10 (20.00%)			42	95 (17.86%)	81 (23.21%)			176
High school or technical secondary school	6 (7.14%)	11 (18.03%)	4 (8.00%)			21	68 (12.78%)	53 (15.19%)			121
Junior college	19 (22.62%)	12 (19.67%)	8 (16.00%)			39	104 (19.55%)	70 (20.06%)			174
Bachelor’s degree or higher	33 (39.29%)	32 (52.46%)	28 (56.00%)			93	265 (49.81%)	145 (44.13%)			410
Occupation:				10.244	0.115				3.275	0.351	
Agricultural industry and traditional services	20 (23.81%)	18 (29.51%)	8 (16.00%)			46	161 (30.26%)	99 (28.37%)			260
Public institution	6 (7.14%)	10 (16.39%)	8 (16.00%)			24	69 (12.97%)	39 (11.17%)			108
Pharmaceutical, financial and high-tech industries	12 (14.29%)	12 (19.67%)	12 (24.00%)			36	105 (19.74%)	61 (17.48%)			166
Other	46 (54.76%)	21 (34.43%)	22 (44.00%)			89	197 (37.03%)	150 (42.98%)			347
Family annual income:				9.456	0.150				12.029	0.007	
< 100,000 yuan	34 (40.48%)	14 (22.95%)	11 (22.00%)			59	110 (20.68%)	101 (28.94%)			211
100,000–300,000 yuan	34 (40.48%)	30 (49.18%)	22 (44.00%)			86	226 (42.48%)	153 (43.84%)			379
300,000–500,000 yuan	9 (10.71%)	12 (19.67%)	10 (20.00%)			31	95 (17.86%)	47 (13.47%)			142
> 500,000 yuan	7 (8.33%)	5 (8.20%)	7 (14.00%)			19	101 (18.98%)	48 (13.75%)			149
Type of school:				3.160	0.206				2.183	0.140	
urban schools	18 (21.43%)	21 (34.43%)	15 (30.00%)			54	176 (33.08%)	99 (28.37%)			275
suburban schools.	66 (78.57%)	40 (65.57%)	35 (70.00%)			141	356 (66.92%)	250 (71.63%)			606
Number of children:				10.182	0.030				12.380	0.002	
1	35 (41.67%)	39 (63.93%)	22 (44.00%)			96	272 (51.13%)	145 (41.55%)			417
2	42 (50.00%)	21 (34.43%)	27 (54.00%)			90	240 (45.11%)	176 (50.43%)			416
> 2	7 (8.33%)	1 (1.64%)	1 (2.00%)			9	20 (3.76%)	28 (8.02%)			48
Number of daughters:				6.456	0.124				3.717	0.155	
1	55 (65.48%)	51 (83.61%)	38 (76.00%)			144	408 (76.69%)	251 (71.92%)			659
2	27 (32.14%)	9 (14.75%)	11 (22.00%)			47	121 (22.74%)	93 (26.65%)			214
> 2	2 (2.38%)	1 (1.64%)	1 (2.00%)			4	3 (0.56%)	5 (1.43%)			8
Knowledge literacy:				9.581	0.048				17.513	< 0.001	
high knowledge literacy	6 (8.00%)	9 (14.75%)	13(26.00%)			28	112 (21.05%)	49 (14.04%)			161
moderate knowledge literacy	30 (40.00%)	28 (45.90%)	17 (34.00%)			84	322 (60.53%)	196 (56.16%)			518
low knowledge literacy	39 (52.00%)	24 (39.34%)	20 (40.00%)			83	98 (18.42%)	104 (29.80%)			202

This study was approved by the Ethics Committee of Minhang District Maternity and Child Health Hospital (Approval No. Min-Fuyou Ethics Review [2024] HS-02).

### Survey tools

The survey comprised three sections. The first section included ten demographic items: gender, age, nationality, marital status, education, occupation, family income, the daughter’s school type, number of children, and number of daughters.

The second section assessed parents’ willingness to vaccinate their daughters against HPV, along with their underlying reasons. Eleven reasons for unwillingness (Fig. 2) and eleven reasons for willingness (Fig. 5) were identified based on a literature review, expert consultation, and qualitative interviews. Participants rated the importance of each reason on a five-point Likert scale.

The third section evaluated knowledge literacy across four domains: cervical cancer, HPV, HPV vaccination, and cervical cancer screening. Total knowledge scores were categorized into high (top 15%), moderate (middle 60%), and low (bottom 25%) groups. The key item sets included the following:

Cervical Cancer Knowledge (12 items): This section began with the question, “Have you heard of cervical cancer?” Respondents who answered “yes” proceeded to questions about the accuracy of relevant statements (e.g., “Cervical screening detects cancer early”) and symptom identification (e.g., “irregular vaginal bleeding,” “nausea and vomiting”). These items were adapted from the Cervical Cancer Knowledge Scale (CCKS), which was originally developed and validated among Canadian women [21].

HPV Knowledge (19 items): This section began with “Have you heard of HPV?” Participants who responded affirmatively were asked to evaluate the accuracy of statements such as “HPV infection is rare” and “HPV infection has obvious symptoms.”

HPV Vaccine Knowledge (10 items): After the question “Have you heard of the HPV vaccine?”, participants assessed statements such as “The HPV vaccine only requires one dose” and “The HPV vaccine can prevent all sexually transmitted infections.” These items were adapted from a Canadian HPV knowledge scale designed for parents [22].

Cervical Cancer Screening Knowledge (12 items): This section started with “Have you heard of cervical cancer screening?” Respondents who answered “yes” proceeded to questions about the accuracy of statements (e.g., “Screening generally starts at age 25,” “Women without a sexual history can begin screening at age 30”). These items were adapted from a knowledge survey of Thai women [23].

Participants responded with “correct,” “incorrect,” or “unsure,” and one point was awarded for each correct response. The questionnaire was reviewed and localized by two clinical experts and demonstrated high reliability (Cronbach’s α values were 0.948 for cervical cancer, 0.961 for HPV, 0.885 for HPV vaccine, and 0.950 for screening; the overall α was 0.965). A pilot study was conducted to confirm the face validity of the questionnaire [17].

### Sample size calculation

The sample size for this study was calculated using the cross-sectional survey sample size estimation formula $n\;=\frac{Z_{1-\alpha/2}^2}{\delta^2}P(1-P)\;(\alpha=0.05,\;Z_{1-\alpha/2}=1.96,\;\delta=5\%).$ Based on a recent meta-analysis conducted in China, the estimated proportion (P) of parents willing to vaccinate their children against HPV was 61.0% [18]. Accordingly, the minimum required sample size was 366. To account for potential non-response and other uncontrollable factors, the target sample size was increased to 1,076.

### Survey method

A convenience sampling method was used to recruit participants. The study selected seven schools in the Minhang District of Shanghai, comprising three urban and four suburban schools. As of the end of 2022, Minhang had a permanent population of 2.6888 million, accounting for 10.86% of Shanghai’s total population and ranking second among the city’s 16 districts [24], thereby making Minhang a highly representative area for this research. The survey targeted parents of girls enrolled in the fourth grade of elementary school to the second year of middle school.

Data collection was conducted via the Questionnaire Star online platform, with participants completing the survey by scanning a Quick Response (QR) code. Healthcare professionals from the maternal and child health hospital provided training to community health directors and school student affairs personnel, who subsequently distributed and explained the survey in schools. Questionnaires were collected anonymously to ensure participant confidentiality, and strict quality control procedures were implemented throughout the data collection process.

### Statistical analysis

All statistical analyses were performed using IBM SPSS Statistics for Windows, version 26 (IBM Corporation, Armonk, NY, USA). Continuous variables were presented as means ± standard deviations (SDs), while categorical variables were summarized as frequencies and percentages.

To explore the reasons and characteristics associated with parents’ willingness or unwillingness to vaccinate their daughters against HPV, a two-step cluster analysis was conducted based on 11 predefined reasons. The analysis employed the log-likelihood distance measure and Schwarz’s Bayesian Information Criterion (BIC) for model selection. Cluster quality was assessed using the silhouette coefficient (range: − 1 to 1), with higher values indicating better-defined cluster separation. This method facilitates the identification of naturally occurring empirical clusters of individuals with similar response patterns [25].

Chi-square tests were used to assess differences in categorical variables, while the Kruskal–Wallis test was applied for skewed continuous variables, with Bonferroni correction applied for multiple comparisons. Multinomial and binary logistic regression analyses were conducted to quantify associations, and results were reported as odds ratios (ORs) with corresponding 95% confidence intervals (CIs). All statistical tests were two-sided, with a significance threshold of *p* < 0.05.

## Results

### Descriptive analyses of clustering results

#### Unwilling groups

**Fig. 1:**
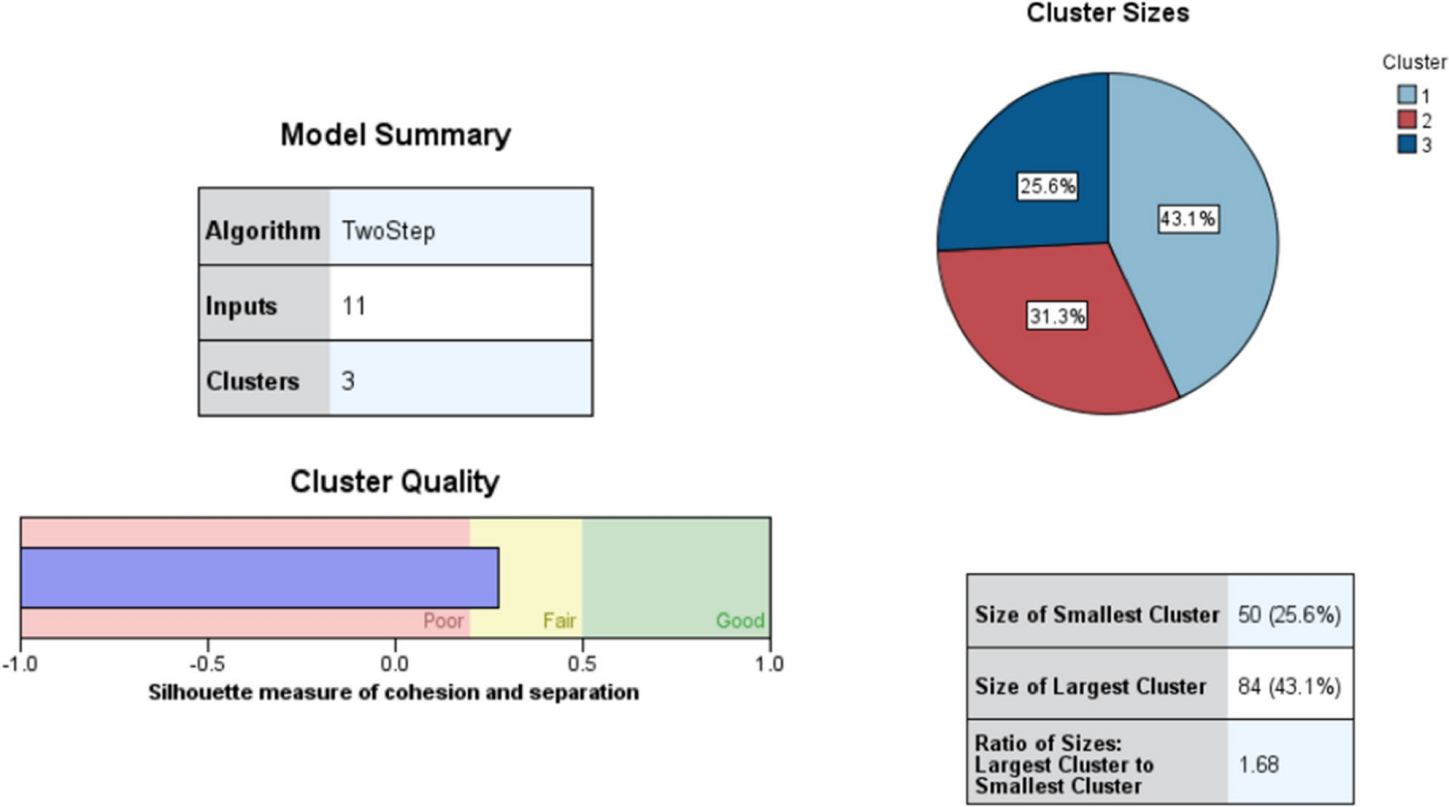
Two-step cluster analysis of unwilling parents

The two-step cluster analysis identified three distinct clusters among the 195 parents who expressed opposition to HPV vaccination for their daughters. The average silhouette coefficient for the model was 0.3, indicating fair cluster quality. The largest cluster included 84 parents (43.1%), while the smallest comprised 50 parents (25.6%). The cluster size ratio (largest to smallest) was 1.68. Details on cluster quality are presented in Fig. 1.

**Fig. 2:**
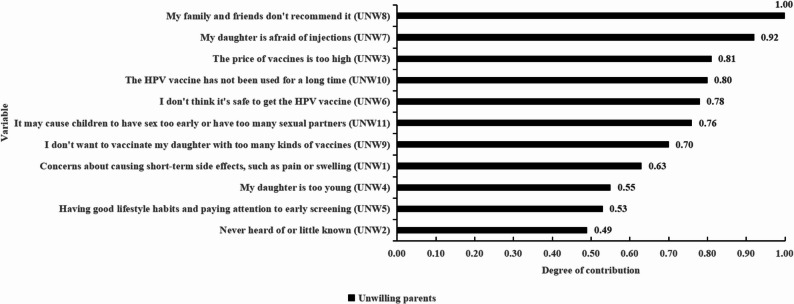
Contribution of reasons for unwillingness in the cluster analysis

Figure 2 illustrates the weight of each reason for unwillingness in the clustering procedure, with values scaled from 0 to 1 to reflect the variable’s contribution to distinguishing empirical groups. Higher values indicate greater discriminative power. The item “My family and friends do not recommend it” had the highest relevance score (1.00), making it the most influential factor in cluster formation. In contrast, the reason “Never heard of or little known” demonstrated the lowest discriminative capacity.

**Fig. 3:**
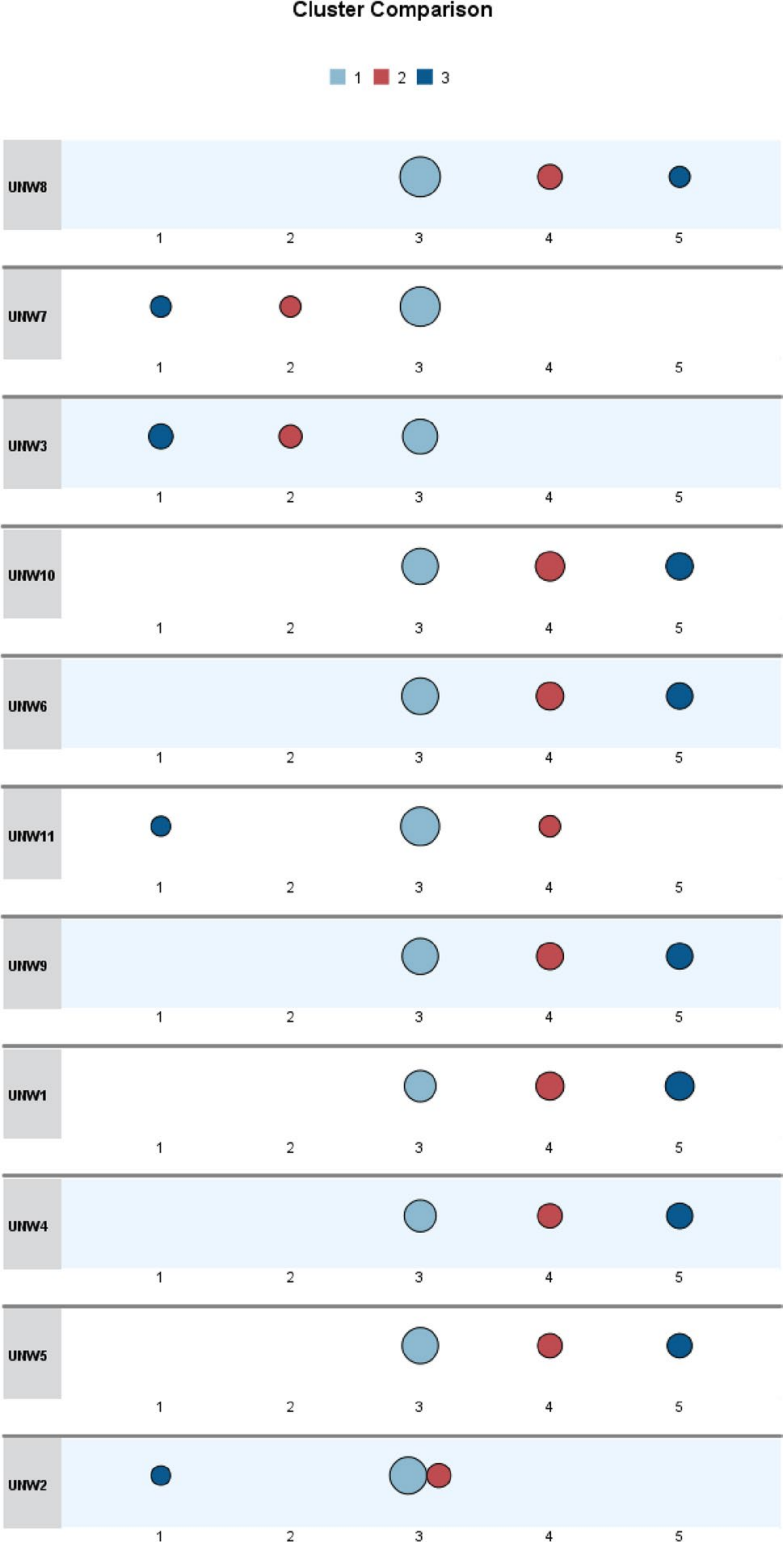
Comparison of characteristics across clusters of unwilling parents

Figure 3 compares the characteristics of the three clusters of parents who were unwilling to vaccinate their daughters against HPV. Cluster 1 (light blue) generally maintained a neutral stance toward the reasons listed in the questionnaire. Cluster 2 (red) tended to agree with most reasons, while Cluster 3 (dark blue) expressed stronger opposition, primarily citing concerns about vaccine safety and the vaccine’s relatively short history.

Based on the interpretation of the patterns shown in the visualized cluster results, descriptive labels were assigned to the three clusters to reflect their distinct characteristics and facilitate reference in subsequent analysis. The cluster labels and their corresponding features are summarized in Table 2.

**Table 2:** Description of cluster results for unwilling groups

Clustering Group	Group Name	Group Characteristics	*N* (%)
cluster 1	Moderate and Hesitant type	Most individuals in this cluster rated the majority of reasons as moderately important (score = 3). They expressed moderate concerns about factors such as vaccine price, recommendations from family and friends, and general vaccine information. Their responses appeared balanced, without strong tendencies toward any specific type of concern, reflecting a generally hesitant and undecided attitude toward HPV vaccination.	84(43.08)
cluster 2	Social-Norm Sensitive type	This group showed low sensitivity (score = 2) to individual-level concerns such as the cost of the vaccine or fear of injections. However, they were more responsive (score = 4) to issues related to social norms and values, including family and peer influence, vaccine safety, and potential implications for sexual behavior. This suggests a heightened sensitivity to external norms and perceived social risks.	61(31.28%)
cluster 3	Trust-Critical type	Individuals in this cluster demonstrated strong concerns (score = 5) regarding trust in the vaccine, including its safety and duration of use. In contrast, they were largely unconcerned (score = 1) about factors such as pain from injections, cost, or moral concerns related to sexual behavior. This pattern indicates a critical stance toward the scientific credibility and safety of the HPV vaccine, reflecting a significant trust deficit.	50(25.64)

#### Willing groups

**Fig. 4:**
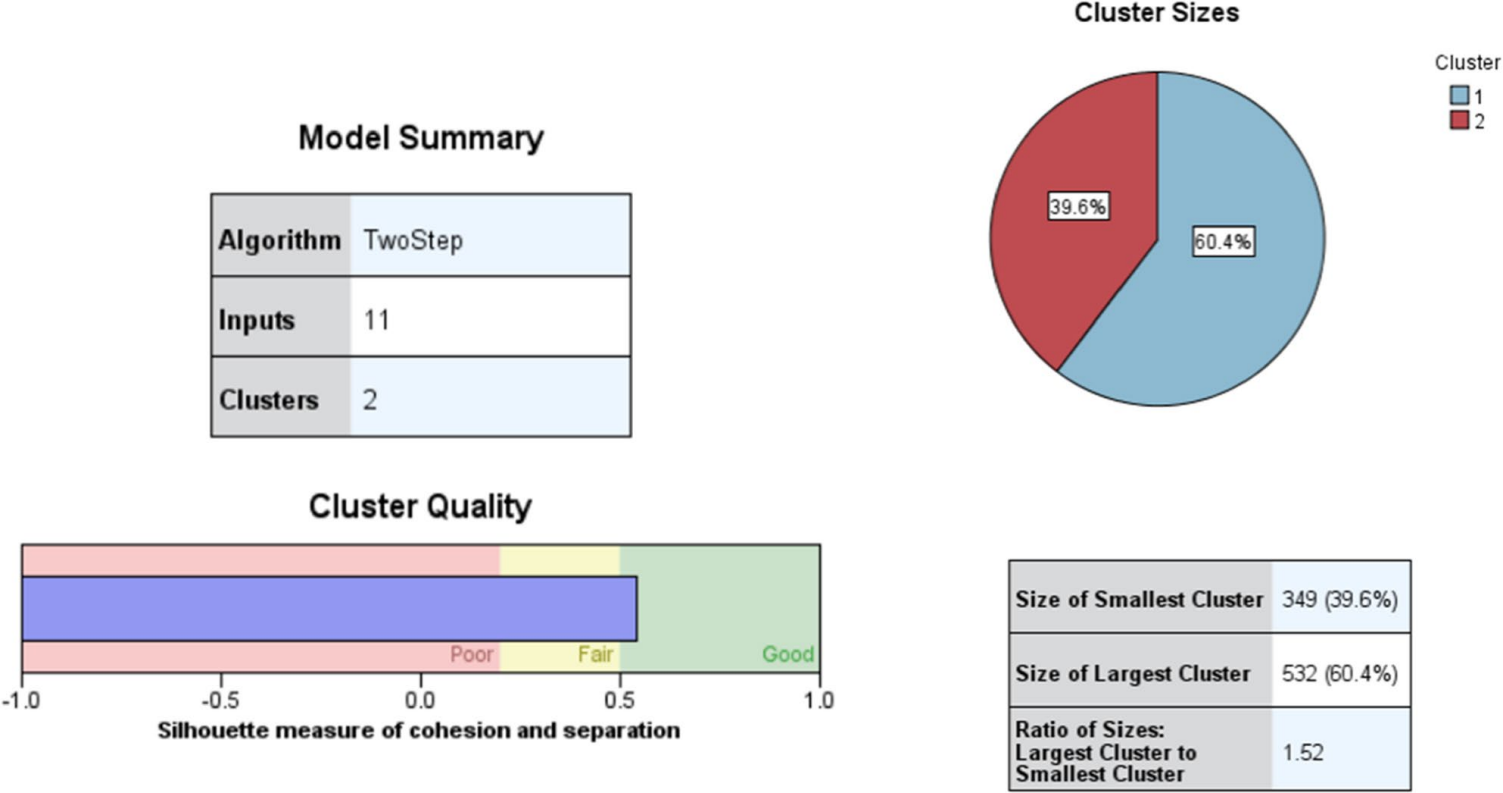
Two-step cluster analysis of willing parents

The two-step cluster analysis identified the two-cluster model as the optimal solution for parents who were willing to vaccinate their daughters, demonstrating the highest distance ratio (1.52) and a favorable cohesion and separation measure (silhouette coefficient = 0.5). This model included 881 parents (Fig. 4).

**Fig. 5:**
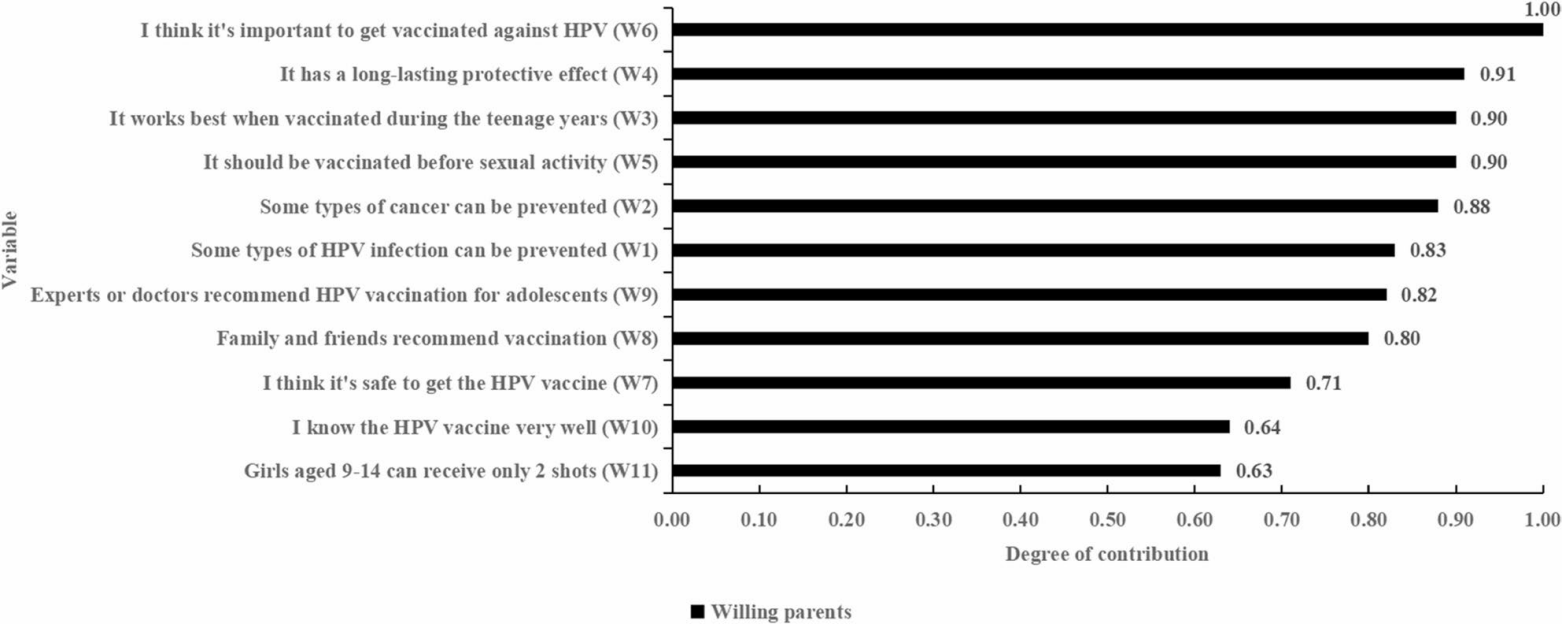
Contribution of reasons for willingness in the cluster analysis

Figure 5 illustrates the weight of each reason for willingness in the clustering analysis. The item “I think it is important to get vaccinated against HPV” had the highest weight (1.00), indicating it was the most influential variable. In contrast, “Girls aged 9–14 can receive only 2 shots” had the lowest discriminative power.

**Fig. 6:**
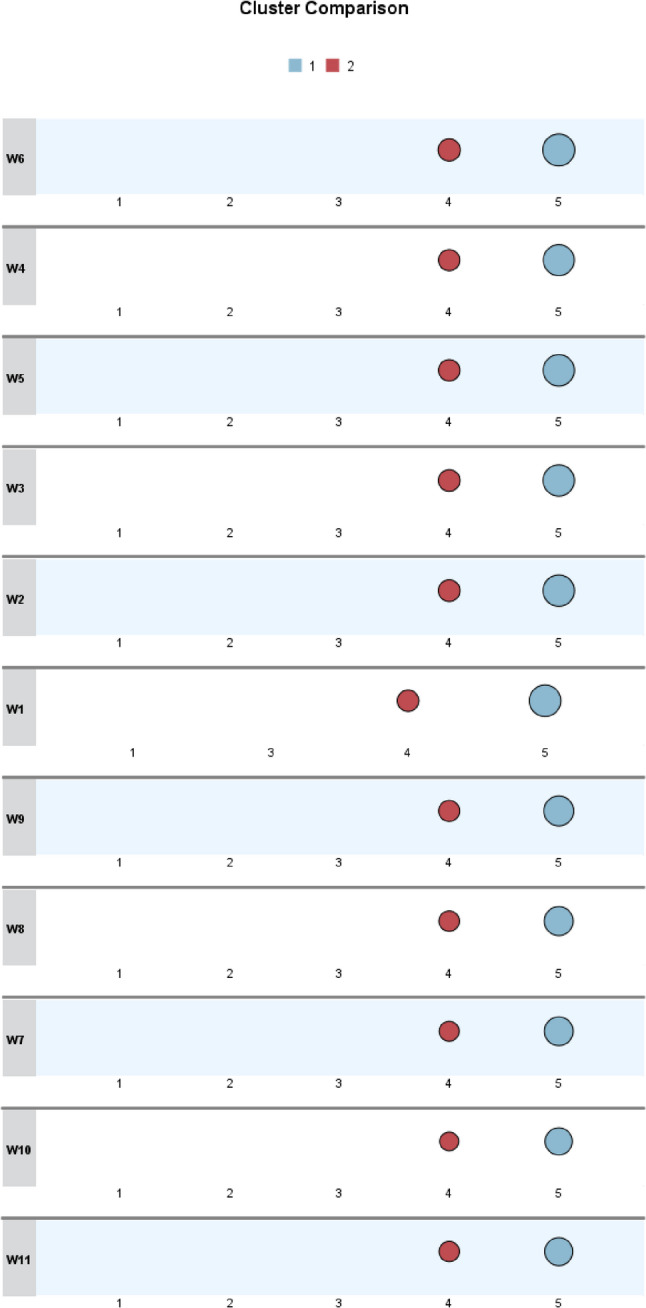
Comparison of characteristics across clusters of willing parents

Figure 6 presents a comparison of the characteristics between the two clusters of parents willing to vaccinate their daughters. Cluster 1 (light blue) assigned the highest possible importance rating (5) to all listed reasons, whereas Cluster 2 (red) rated the reasons slightly lower, generally assigning ratings of 4.

To reflect the distinguishing features identified through clustering, the two clusters were assigned descriptive labels for ease of reference. Detailed names and defining characteristics of the clusters are provided in Table 3.

**Table 3:** Description of cluster results for willing groups

Clustering Group	Group Name	Group Characteristics	*N* (%)
cluster 1	Highly Affirmative type	Nearly all reasons were rated as 5 (very important), with consistently large circle sizes indicating strong consensus. High-contribution variables such as W6, W4, W3, and W5 all received the highest rating (5) with the largest circles. This group represents a highly affirmative population who exhibit strong confidence and recognition of the scientific value, safety, timing, and effectiveness of the HPV vaccine. Their attitude is firm and highly supportive.	532(55.85)
cluster 2	Moderately Affirmative type	Most reasons were rated as 4 (important). While the high-contribution variables also received a score of 4, the circle sizes were slightly smaller compared to the other group, indicating a relatively lower level of agreement. This group demonstrates a generally supportive but somewhat reserved attitude toward HPV vaccination. They acknowledge its importance and express support, yet their conviction appears less strong, possibly due to lingering uncertainty or limited knowledge.	349(44.15)

### Associations between individual characteristics and clustering results

Chi-square tests revealed significant differences among the HPV vaccine refusal clusters in educational attainment, number of children, and knowledge literacy (*p* < 0.05). Specifically, within the Moderate and Hesitant type, individuals holding a bachelor’s degree or higher constituted the largest proportion (39.29%); however, this group also exhibited the highest proportion of individuals with a junior high school or below among the three types (30.95%). In the Social-Norm Sensitive type, more than half of the respondents had a bachelor’s degree or higher (52.46%), while only 9.84% had a junior high school or below—the lowest proportion among all types. Similarly, in the Trust-Critical type, over half of the individuals (56.00%) had a bachelor’s degree or higher (56.00%).

Among respondents classified as the Social-Norm Sensitive type, the majority had one child (63.93%). In contrast, most respondents in the Moderate and Hesitant type and the Trust-Critical type had two children (50.00% and 54.00%, respectively). Although the overall proportion of individuals with three or more children was relatively low, it was slightly higher in the Moderate and Hesitant type (8.33%).

The Moderate and Hesitant type exhibited the highest proportion of individuals with low knowledge literacy among the three groups (52%), suggesting that their vaccine refusal may stem from a lack of information or cognitive biases. The Trust-Critical type had the highest proportion of individuals with high knowledge literacy (26%), although those with low literacy still accounted for 40%, indicating a potential polarization within this group. Some individuals may reject vaccination not due to a lack of knowledge, but rather as a result of rational critiques based on high scientific literacy and concerns regarding vaccine safety; others may refuse due to misinformation. The distribution of knowledge literacy within the Social-Norm Sensitive type was relatively balanced, with the largest proportion falling into the medium literacy category (45.9%), suggesting that their attitudes may be more influenced by social factors (e.g., peer opinions or prevailing recommendations) than by individual knowledge levels.

Details are presented in Table 1.

Table 4 presents differences in knowledge literacy scores across four dimensions—cervical cancer, HPV, HPV vaccine, and cervical cancer screening—among individuals with varying levels of willingness to receive vaccination. Overall, individuals who expressed willingness to be vaccinated demonstrated higher levels of knowledge literacy than those who were unwilling, suggesting that knowledge may play a critical role in shaping vaccination intention.

**Table 4:** Relationship between knowledge literacy and clustering results, mean (SD)

Knowledge literacy score composition	Unwilling parents (*N* = 195)				Willing parents (*N* = 881)		
	Moderate and Hesitant type	Social-Norm Sensitive type	Trust-Critical type	Kruskal‒Wallis H	Highly Affirmative type	Moderately Affirmative type	Kruskal‒Wallis H
Cervical cancer	6.36(3.21)	7.48(3.02)^a^	7.04(2.72)	7.102*	7.84(2.38)	7.16(2.74)	17.659**
HPV	4.70(5.49)	6.38(6.04)	7.28(6.51)	5.921	8.65(5.25)	6.89(5.67)	18.962**
HPV vaccine	3.70(3.26)	4.00(3.04)	4.34(3.33)	1.538	5.40(2.79)	4.73(2.94)	11.028**
Cervical cancer screening	2.82(3.36)	3.56(3.45)	3.66(3.68)	2.558	4.42(3.38)	3.88(3.46)	4.753*

a. Significantly different from the Moderate and Hesitant type (post hoc test, *p* < 0.05).

* Significant at *p* ≤ 0.05, ** significant at *p* ≤ 0.001.

For cervical cancer knowledge, the Kruskal–Wallis H test identified statistically significant differences within both the unwilling group (H = 7.102, *p* < 0.05) and the willing group (H = 17.659, *p* < 0.001), indicating variability in scores among clusters within each group. Post hoc comparisons revealed a significant difference between the Moderate and Hesitant type and the Social-Norm Sensitive type (*p* = 0.023), with the latter scoring higher. No significant differences were observed among the remaining types (*p* > 0.05), suggesting that individuals in the Moderate and Hesitant type possessed comparatively limited knowledge about cervical cancer.

In contrast, knowledge scores related to HPV, HPV vaccination, and cervical cancer screening showed statistically significant differences across clusters within the willing group, whereas no significant differences were found across clusters within the unwilling group.

### Regression analysis

As shown in Table 5, a multinomial logistic regression analysis was conducted to examine the factors associated with participants’ classification into different refusal clusters, using the Moderate and Hesitant type as the reference category. The results indicated that participants’ educational attainment significantly distinguished individuals in the Social-Norm Sensitive type from those in the reference group. Specifically, participants with a junior high school or below were significantly less likely to be classified as Social-Norm Sensitive compared to those with a bachelor’s degree or higher (OR = 0.288, 95% CI: 0.101–0.826, *p* = 0.021), suggesting that this type is more likely to include highly educated parents.

**Table 5:** Multinomial logistic regression analysis of the clustering results for unwilling parents

unwilling groups^a^		b	SE	Wald^2^	*p*-value	OR(95%CI)
Social-Norm Sensitive type	Intercept	−0.273	1.241	0.048	0.826	
	Number of children:					
	1	1.524	1.131	1.817	0.178	4.590 (0.501–42.084)
	2	0.636	1.145	0.308	0.579	1.888 (0.200–17.801)
	> 2^b^	.	.	.	.	.
	Highest educational attainment:					
	Junior high school or below	−1.244	0.537	5.366	0.021	0.288 (0.101–0.826)
	High school or technical secondary school	0.881	0.589	2.236	0.135	2.414 (0.760–7.666)
	Junior college	−0.358	0.458	0.610	0.435	0.699 (0.285–1.715)
	Bachelor’s degree or higher^b^	.	.	.	.	.
	Knowledge literacy:					
	low knowledge literacy	−1.079	0.625	2.982	0.084	0.340 (0.100–1.157)
	moderate knowledge literacy	−0.995	0.619	2.582	0.108	0.370 (0.110–1.244)
	high knowledge literacy^b^	.	.	.	.	.
Trust-Critical type	Intercept	−0.172	1.222	0.020	0.888	
	Number of children:					
	1	1.311	1.146	1.307	0.253	3.708 (0.392–35.058)
	2	1.226	1.141	1.156	0.282	3.408 (0.364–31.862)
	> 2^b^	.	.	.	.	.
	Highest educational attainment:					
	Junior high school or below	−0.653	0.479	1.860	0.173	0.521 (0.204–1.330)
	High school or technical secondary school	−0.088	0.710	0.015	0.901	0.915 (0.227–3.683)
	Junior college	−0.558	0.506	1.214	0.271	0.573 (0.212–1.544)
	Bachelor’s degree or higher^b^	.	.	.	.	.
	Knowledge literacy:					
	low knowledge literacy	−1.409	0.582	5.854	0.016	0.244 (0.078–0.765)
	moderate knowledge literacy	−1.594	0.593	7.231	0.007	0.203 (0.064–0.649)
	high knowledge literacy^b^	.	.	.	.	.

a. The reference category is Moderate and Hesitant type

b. This parameter is set as a reference value

Knowledge literacy significantly differentiated individuals in the Trust-Critical type from those in the reference group. Compared to individuals with high knowledge literacy, those with low (OR = 0.244, 95% CI: 0.078–0.765, *p* = 0.016) and moderate knowledge literacy (OR = 0.203, 95% CI: 0.064–0.649, *p* = 0.007) were significantly less likely to be classified as the Trust-Critical type. This finding indicates that individuals with high knowledge literacy are more likely to be assigned to the Trust-Critical type, whereas those with low or moderate knowledge literacy are more likely to fall into the Moderate and Hesitant type.

As shown in Table 6, a binary logistic regression analysis was conducted among parents who expressed willingness to vaccinate their daughters against HPV, using the Moderately Affirmative type as the reference category. The number of children and knowledge literacy level significantly distinguished the two groups. Specifically, compared to parents with one child, those with more than two children were significantly more likely to be classified as the Highly Affirmative type (OR = 2.228, 95% CI: 1.199–4.141, *p* = 0.011), indicating that parents with more children tended to be more supportive of HPV vaccination for their daughters.

**Table 6:** Binary logistic regression analysis of the clustering results for willing parents

	b	SE	Wald^2^	*p*-value	OR(95%CI)
Family annual income:					
< 100,000 yuan^a^	.	.	5.374	0.146	.
100,000–300,000 yuan	−0.153	0.179	0.729	0.393	0.858 (0.605–1.219)
310,000–500,000 yuan	−0.432	0.232	3.486	0.062	0.649 (0.412–1.022)
> 500,000 yuan	−0.434	0.231	3.509	0.061	0.648 (0.412–1.020)
Number of children:					
1^a^	.	.	7.971	0.019	.
2	0.269	0.146	3.388	0.066	1.309 (0.983–1.743)
> 2	0.801	0.316	6.416	0.011	2.228 (1.199–4.141)
Knowledge literacy level:					
low knowledge literacy^a^	.	.	10.897	0.004	.
moderate knowledge literacy	−0.459	0.171	7.174	0.007	0.632 (0.451–0.884)
high knowledge literacy	−0.707	0.229	9.509	0.002	0.493 (0.315–0.773)
Constant	0.002	0.201	0.000	0.993	0.584

a. This parameter is set as a reference value.

Parents with moderate knowledge literacy (OR = 0.632, 95% CI: 0.451–0.884, *p* = 0.007) and high knowledge literacy (OR = 0.493, 95% CI: 0.315–0.773, *p* = 0.002) were significantly less likely to be classified as the Highly Affirmative type compared to those with low knowledge literacy, suggesting a greater likelihood of classification into the Moderately Affirmative type. This finding indicates that individuals with higher levels of knowledge literacy may exhibit a more cautious or nuanced stance toward HPV vaccination than those with lower literacy levels.

## Discussion

Of the 1,195 valid questionnaires, 119 parents (9.96%) reported that their daughters had received the HPV vaccine or were scheduled to receive it. A prior Chinese study reported that although 32.6% of parents intended to vaccinate, only 16.4% successfully scheduled vaccination [26]; this gap underscores the need to examine willingness to vaccinate and the characteristics of parents with unvaccinated girls.

Among the 1,076 parents of 9–14-year-old girls who had not received the HPV vaccine, 81.88% reported willingness to vaccinate, whereas 18.12% reported unwillingness. This proportion is comparable to the 85.1% reported in Poland [27] and is substantially higher than the 61.0% (95% CI: 53.5–68.3%) estimated in a recent meta-analysis of Chinese parents [18] and the 21.9% reported in a 2022 Japanese survey [28]. In contrast, willingness was higher in Bangladesh, where most married women reported an intention to vaccinate themselves (urban: 93.9%, rural: 99.4%) and their daughters (urban: 91.8%, rural: 99.2%) [29]. The high proportion of female respondents in this study (91.54%) may reflect mothers’ central role in vaccination decision-making [30]. Similarly, a U.S. study noted that because mothers are typically the primary decision-makers for adolescent vaccination, identifying persistent knowledge gaps and misconceptions among parents is critical to designing effective interventions [31].

### Vaccine refusal is a structurally complex phenomenon encompassing multiple, distinct forms of refusal

The three refusal types identified through two-step cluster analysis among parents who declined HPV vaccination for their 9–14-year-old daughters (Moderate and Hesitant, Social-Norm Sensitive, and Trust-Critical) differed significantly, indicating that vaccine refusers are not a homogeneous group. Although this typology is novel, it is consistent with the logic of prior research [32]. Notably, these results challenge the conventional assumption that vaccine hesitancy is primarily attributable to insufficient knowledge: parents in the Trust-Critical type demonstrated high knowledge literacy yet still refused vaccination, suggesting a pattern of informed distrust. Collectively, these findings underscore the need for tailored interventions; a one-size-fits-all approach to health education or science communication is unlikely to be effective across refuser clusters.

### Trust-Critical type: rational refusers with high knowledge but low trust

This group is characterized by higher educational attainment and the highest proportion of individuals with high knowledge literacy. Nevertheless, some parents still refuse HPV vaccination for their daughters, reflecting rational skepticism. These findings suggest that vaccine distrust cannot be attributed solely to low educational attainment, a pattern consistent with evidence from high-income countries where negative vaccine attitudes persist despite broad access to education and healthcare [20].

The 3 C model—confidence, complacency, and convenience—provides a useful framework for interpreting this phenomenon [33]. Lack of confidence may stem from concerns about vaccine safety or skepticism regarding information sources. Complacency refers to a low perceived risk of HPV infection, whereas constraints on convenience include time limitations or difficulties accessing vaccination services. In this study, refusal within the Trust-Critical type appears to be driven primarily by systemic distrust directed at government, pharmaceutical companies, and/or vaccines themselves. Accordingly, an effective response requires not only persuasive communication but also sustained efforts to rebuild public trust.

In contrast to a previous study [34], communication and recommendations from healthcare professionals alone appear insufficient to restore trust; trust remained limited [31]. This suggests that providers must themselves have confidence in vaccine safety and efficacy [35]. However, hesitancy has also been documented among healthcare providers, including those who administer vaccines. For example, up to 60% of French general practitioners reported concerns about vaccine safety [36], and nearly one-quarter of those with daughters reported having no intention to vaccinate [37].

Although provider recommendations can increase the likelihood of parental acceptance [38], 60.6% of parents of unvaccinated adolescents still reported resisting HPV vaccination even after receiving such advice [39]. Moreover, while healthcare professionals are the most trusted source of vaccine information for many people, evidence indicates that some parents still question the objectivity of the information provided by clinicians [40].

To address this gap, governments, healthcare systems, and vaccine developers should improve the transparency and clarity of information regarding vaccine safety and effectiveness. These efforts may reduce misinformation and strengthen trust among both the public and healthcare professionals. In addition, long-term follow-up studies should be expanded to provide more robust evidence on the benefits and risks of vaccination. Ethically, prioritizing vaccination coverage while neglecting transparent and comprehensive information disclosure may be regarded as discriminatory on the basis of healthy, innate biological characteristics; such an approach may contravene ethical norms and may be difficult to justify on ethical grounds [41].

### Moderate and Hesitant type: a combined group characterized by information deficits and uncertainty

This group is characterized by pronounced disparities in educational attainment and a predominance of low knowledge literacy, indicating a substantial need for targeted science communication. Although members of this group may not categorically reject vaccines, their hesitancy is more likely to reflect limited understanding and a low perceived risk of infection. This pattern is consistent with prior evidence that parents with limited health literacy are more likely to exhibit vaccine hesitancy about vaccinating their children [42–44].

The Health Belief Model (HBM) provides a useful framework for interpreting this type [33]. Under HBM, limited knowledge about HPV and its vaccine among individuals in the Moderate and Hesitant type may contribute to underestimation of their daughters’ infection risk, thereby weakening perceived susceptibility. In addition, limited awareness of HPV-related outcomes (e.g., cervical cancer) may reduce perceived severity. Collectively, these cognitive gaps may diminish motivation to vaccinate daughters against HPV.

The HBM therefore highlights the importance of targeted health education and risk communication for this type. Given their significantly lower cervical cancer knowledge scores than those of the Social-Norm Sensitive type, interventions should prioritize improving awareness of cervical cancer and related health consequences, rather than focusing solely on vaccine promotion. Specifically, efforts should strengthen understanding of the severity of cervical cancer and the preventability of HPV-related disease.

### Social-Norm Sensitive type: highly educated and influenced by social norms

This group typically has only one child and places greater weight on mainstream vaccination advice, potentially because parents may perceive higher stakes in decision-making for an only child. Knowledge literacy in this type is relatively balanced, and overall educational attainment is comparatively high, suggesting that higher education may be associated with greater sensitivity to social norms. Logistic regression further suggests that sensitivity to social norms may vary by educational background, with lower educational attainment potentially reducing susceptibility to normative influences that shape vaccine refusal intentions.

For this type, vaccine awareness may be strengthened through indirect, socially embedded approaches that leverage social networks and community influence. Potential strategies include peer-led guidance, celebrity endorsements, and messaging through mainstream media. However, existing evidence indicates that peers and media—although important sources of health information—may also contribute to vaccine hesitancy and the dissemination of misinformation [31]. Social media platforms in particular have been identified as prominent sources of confusion and distrust. For example, an analysis of Facebook posts about the HPV vaccine found that negative content predominated: barriers to vaccination were mentioned in 47.1% of posts, whereas benefits appeared in only 19.8%; overall, 45.0% of posts conveyed negative sentiment [45]. These findings underscore the need for strengthened governance and evidence-based communication on social media to mitigate misinformation about the HPV vaccine.

### Limitations

This study has several limitations. First, convenience sampling may have introduced selection bias. Second, data were collected from a single district in Shanghai, which may limit the generalizability of the findings to other settings. Third, the cross-sectional design precludes causal inference.

In addition, among families with more than one child, information on parents’ HPV vaccination decisions for other children was not collected. Although the present study focused on parental attitudes, knowledge, and sociodemographic characteristics related to HPV vaccination decisions for daughters aged 9–14 years, HPV vaccination attitudes or decisions regarding older children were not assessed. Such information could provide additional context for analysis and interpretation and should be considered in future research.

Finally, this study examined parental decision-making regarding HPV vaccination for girls only, reflecting the current policy focus on female vaccination in China. Parental attitudes toward HPV vaccination for boys were not assessed and may differ. As HPV vaccination policies for males continue to evolve, future research could consider including boys to provide a more comprehensive understanding of gender-specific parental decision-making.

## Conclusion

**Fig. 7:**
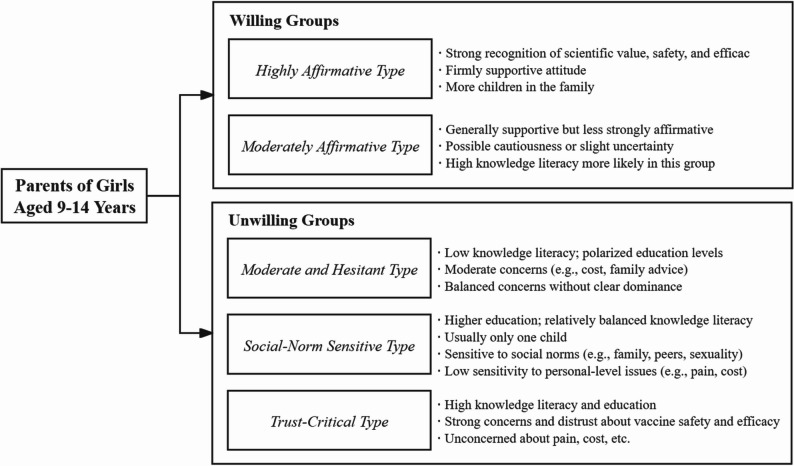
Key characteristics of parents willing and unwilling to vaccinate their daughters against HPV

This study identified three distinct clusters among parents unwilling to vaccinate their 9–14-year-old daughters, confirming that HPV vaccine refusal is heterogeneous. The Moderate and Hesitant type was characterized by low knowledge literacy and limited awareness of HPV-related risks, suggesting a need for education that emphasizes disease severity. The Trust-Critical type exhibited informed skepticism despite high knowledge literacy, indicating the importance of transparent and credible information. The Social-Norm Sensitive type appeared to be shaped by peer influence and social norms, supporting the use of socially embedded strategies. Additional details are presented in Fig. 7. Overall, these findings underscore the need for tailored interventions that address cluster-specific motivations rather than uniform campaigns.

## Data Availability

The data used in this study were collected by the authors through a self-administered cross-sectional survey. Because the dataset contains potentially sensitive information about participants’ health beliefs and vaccine decisions, it is not publicly available. However, de-identified data and analysis code are available from the corresponding author upon reasonable request for the purpose of academic replication.

## Abbreviations

BIC: Bayesian information criterion
CCKS: Cervical Cancer Knowledge Scale
CI: Confidence intervals
HBM: Health Belief Model
HPV: Human papillomavirus
NIP: National immunization program
OR: Odds ratio
QR: Quick Response
SD: Standard deviations
WHO: World Health Organization

## References

[1] World Health Organization. Human papillomavirus and cancer. Available from: https://www.who.int/news-room/fact-sheets/detail/human-papilloma-virus-and-cancer. Accessed September 26, 2024.

[2] Kamolratanakul S, Pitisuttithum P. Human papillomavirus vaccine efficacy and effectiveness against cancer. Vaccines (Basel). 2021;9:1413. 10.3390/vaccines9121413. 34960159 10.3390/vaccines9121413 PMC8706722

[3] Centers for Disease Control and Prevention. Basic Information about HPV and Cancer. Available from: https://www.cdc.gov/cancer/hpv/basic-information.html. Accessed September 26, 2024.

[4] NATIONAL CANCER INSTITUTE. Cancer Stat Facts. Cervical Cancer. Available from: https://seer.cancer.gov/statfacts/html/cervix.html. Accessed September 26, 2024.

[5] World Health Organization. Human papillomavirus vaccines: WHO position paper (‎2022 update)‎‎. Wkly Epidemiol Rec. 2022;97:645–72. 10.1136/bmj-2022-070135.

[6] Kahn BZ, Reiter PL, Kritikos KI, Gilkey MB, Queen TL, Brewer NT. Framing of National HPV vaccine recommendations and willingness to recommend at ages 9–10. Hum Vaccin Immunother. 2023;19:2172276. 10.1080/21645515.2023.2172276. 36749614 10.1080/21645515.2023.2172276 PMC10012934

[7] World Health Organization. Global strategy to accelerate the elimination of cervical cancer as a public health problem. Available from: https://www.who.int/publications/i/item/9789240014107. Accessed September 26, 2024.

[8] The State Council of the People’s Republic of China. Notice on the issuance of the Action Plan for Accelerating the Elimination of Cervical Cancer (2023–2030). Available from: https://www.gov.cn/zhengce/zhengceku/2023-01/21/content_5738364.htm#:~:text=%E4%B8%BA%E8%B4%AF%E5%BD%BB%E8%90%BD%E5%AE%9E%E3%80%8A%E2%80%9C%E5%81%A5. Accessed September 26, 2024.

[9] National Health Commission of the People’s Republic of China. Reply to Recommendations Nos. 358, 3582 and 5702 of the Second Session of the 14th National People’s Congress. Available from: http://www.nhc.gov.cn/wjw/jiany/202408/4b56b64be631441a9be8d9ffe9a0626f.shtml. Accessed September 26, 2024.

[10] Shanghai Municipal Health Commission. Reply to Proposal No. 0153 of the Fifth Session of the 13th CPPCC. Available from: https://wsjkw.sh.gov.cn/zxtadf/20220530/45d51adf0c4c48d8ac9b617c9c773c41.html. Accessed September 26, 2024.

[11] Wang C, Wang Y, Han B, Zhao TS, Liu B, Liu H, et al. Willingness and SARS-CoV-2 vaccination coverage among healthcare workers in china: a nationwide study. Vaccines (Basel). 2021;9:993. 10.3390/vaccines9090993. 34579230 10.3390/vaccines9090993 PMC8472967

[12] Liu R, Li Y, Wangen KR, Maitland E, Nicholas S, Wang J. Analysis of hepatitis B vaccination behavior and vaccination willingness among migrant workers from rural China based on protection motivation theory. Hum Vaccin Immunother. 2016;12:1155–63. 10.1080/21645515.2015.1123358. 27078191 10.1080/21645515.2015.1123358 PMC4963072

[13] National Academies Press. Health Literacy: A Prescription to End Confusion. Available from: https://www.researchgate.net/publication/303697782_Health_Literacy_A_Prescription_to_End_Confusion. Accessed October 11, 2024. 25009856

[14] Batterham RW, Hawkins M, Collins PA, Buchbinder R, Osborne RH. Health literacy: applying current concepts to improve health services and reduce health inequalities. Public Health. 2016;132:3–12. 10.1016/j.puhe.2016.01.001. 26872738 10.1016/j.puhe.2016.01.001

[15] Lindau ST, Tomori C, Lyons T, Langseth L, Bennett CL, Garcia P. The association of health literacy with cervical cancer prevention knowledge and health behaviors in a multiethnic cohort of women. Am J Obstet Gynecol. 2002;186:938–43. 10.1067/mob.2002.122091. 12015518 10.1067/mob.2002.122091

[16] Sayar MS, Akça MÖ, Hakyemez ISN, Asan A. The impact of health literacy on COVID-19 immunization. Hum Vaccin Immunother. 2023;19:2254539. 10.1080/21645515.2023.2254539. 37814493 10.1080/21645515.2023.2254539 PMC10566376

[17] Kitur H, Horowitz AM, Beck K, Wang MQ. HPV knowledge, vaccine status, and health literacy among university students. J Cancer Educ. 2022;37:1606–13. 10.1007/s13187-021-01997-1. 33768470 10.1007/s13187-021-01997-1

[18] Tan S, Wang S, Zou X, Jia X, Tong C, Yin J, et al. Parental willingness of HPV vaccination in Mainland China: a meta-analysis. Hum Vaccin Immunother. 2024;20:2314381. 10.1080/21645515.2024.2314381. 38385893 10.1080/21645515.2024.2314381 PMC10885179

[19] Smith PJ, Stokley S, Bednarczyk RA, Orenstein WA, Omer SB. HPV vaccination coverage of teen girls: the influence of health care providers. Vaccine. 2016;34:1604–10. 10.1016/j.vaccine.2016.01.061. 26854907 10.1016/j.vaccine.2016.01.061 PMC7285619

[20] Dubé È, Ward JK, Verger P, MacDonald NE. Vaccine hesitancy, acceptance, and anti-vaccination: trends and future prospects for public health. Annu Rev Public Health. 2021;42:175–91. 10.1146/annurev-publhealth-090419-102240. 33798403 10.1146/annurev-publhealth-090419-102240

[21] Haward B, Tatar O, Zhu P, Griffin-Mathieu G, Perez S, Shapiro GK, et al. Development and validation of the cervical cancer knowledge scale and HPV testing knowledge scale in a sample of Canadian women. Prev Med Rep. 2022;30:102017. 10.1016/j.pmedr.2022.102017. 36281348 10.1016/j.pmedr.2022.102017 PMC9587520

[22] Perez S, Tatar O, Ostini R, Shapiro GK, Waller J, Zimet G, et al. Extending and validating a human papillomavirus (HPV) knowledge measure in a national sample of Canadian parents of boys. Prev Med. 2016;91:43–9. 10.1016/j.ypmed.2016.07.017. 27471023 10.1016/j.ypmed.2016.07.017

[23] Khomphaiboonkij U, Sreamsukcharoenchai N, Pitakkarnkul S, Rittiluechai K, Tangjitgamol S. Knowledge of Thai women in cervical cancer etiology and screening. PLoS One. 2023;18:e0286011. 10.1371/journal.pone.0286011. 37200337 10.1371/journal.pone.0286011 PMC10194861

[24] Shanghai Municipal Bureau of Statistics. Shanghai Statistical Yearbook. 2023. Available from: https://tjj.sh.gov.cn/tjnj/20240321/5a35a44acace471f87c75393133fa142.html. Accessed October 11, 2024.

[25] Zhao DT, Yan HP, Liao HY, Liu YM, Han Y, Zhang HP, et al. Using two-step cluster analysis to classify inpatients with primary biliary cholangitis based on autoantibodies: a real-world retrospective study of 537 patients in China. Front Immunol. 2022;13:1098076. 10.3389/fimmu.2022.1098076. 36685575 10.3389/fimmu.2022.1098076 PMC9845730

[26] Wang X, Pan J, Yan B, Zhang R, Yang T, Zhou X. Inequities in human papillomavirus vaccination among children aged 9–14 years old under constrained vaccine supply in China. Int J Equity Health. 2024;23:112. 10.1186/s12939-024-02199-z. 38822383 10.1186/s12939-024-02199-z PMC11141026

[27] Ganczak M, Owsianka B, Korzeń M. Factors that predict parental willingness to have their children vaccinated against HPV in a country with low HPV vaccination coverage. Int J Environ Res Public Health. 2018;15:645. 10.3390/ijerph15040645. 29614733 10.3390/ijerph15040645 PMC5923687

[28] Suzuki Y, Sukegawa A, Ueda Y, Sekine M, Enomoto T, Melamed A, et al. The effect of a web-based cervical cancer survivor’s story on parents’ behavior and willingness to consider human papillomavirus vaccination for daughters: randomized controlled trial. JMIR Public Health Surveill. 2022;8:e34715. 10.2196/34715. 35421848 10.2196/34715 PMC9178460

[29] Islam JY, Khatun F, Alam A, Sultana F, Bhuiyan A, Alam N, et al. Knowledge of cervical cancer and HPV vaccine in Bangladeshi women: a population based, cross-sectional study. BMC Womens Health. 2018;18:15. 10.1186/s12905-018-0510-7. 29325530 10.1186/s12905-018-0510-7 PMC5765714

[30] Runngren E, Eriksson M, Blomberg K. Parents’ reasoning about HPV vaccination in Sweden. Scand J Caring Sci. 2022;36:1113–22. 10.1111/scs.13041. 34672006 10.1111/scs.13041

[31] Walker KK, Owens H, Zimet G. “We fear the unknown": emergence, route and transfer of hesitancy and misinformation among HPV vaccine accepting mothers. Prev Med Rep. 2020;20:101240. 10.1016/j.pmedr.2020.101240. 33294312 10.1016/j.pmedr.2020.101240 PMC7689543

[32] Gopalani SV, Janitz AE, Burkhart M, Campbell JE, Chen S, Martinez SA, White AH, Anderson AS, Pharr SF, Peck JD, Comiford A. HPV vaccination coverage and factors among American Indians in Cherokee Nation. Cancer Causes Control. 2023;34:267–75. 10.1007/s10552-022-01662-y. 36542212 10.1007/s10552-022-01662-y PMC9768789

[33] Tostrud L, Thelen J, Palatnik A. Models of determinants of COVID-19 vaccine hesitancy in non-pregnant and pregnant population: review of current literature". Hum Vaccin Immunother. 2022;18:2138047. 10.1080/21645515.2022.2138047. 36345571 10.1080/21645515.2022.2138047 PMC9746492

[34] Wang H, Jiang Y, Wang Q, Lai Y, Holloway A. The status and challenges of HPV vaccine programme in China: an exploration of the related policy obstacles. BMJ Glob Health. 2023;8:e012554. 10.1136/bmjgh-2023-012554. 37586782 10.1136/bmjgh-2023-012554 PMC10432676

[35] Deml MJ, Buhl A, Notter J, Kliem P, Huber BM, Pfeiffer C, et al. “Problem patients and physicians” failures’: what it means for doctors to counsel vaccine hesitant patients in Switzerland. Soc Sci Med. 2020;255:112946. 10.1016/j.socscimed.2020.112946. 32311515 10.1016/j.socscimed.2020.112946

[36] Killian M, Detoc M, Berthelot P, Charles R, Gagneux-Brunon A, Lucht F, et al. Vaccine hesitancy among general practitioners: evaluation and comparison of their immunisation practice for themselves, their patients and their children. Eur J Clin Microbiol Infect Dis. 2016;35:1837–43. 10.1007/s10096-016-2735-4. 27488435 10.1007/s10096-016-2735-4

[37] Collange F, Fressard L, Pulcini C, Sebbah R, Peretti-Watel P, Verger P. General practitioners’ attitudes and behaviors toward HPV vaccination: a French national survey. Vaccine. 2016;34:762–8. 10.1016/j.vaccine.2015.12.054. 26752063 10.1016/j.vaccine.2015.12.054

[38] Sonawane K, Zhu Y, Lin YY, Damgacioglu H, Lin Y, Montealegre JR, et al. HPV vaccine recommendations and parental intent. Pediatrics. 2021;147:e2020026286. 10.1542/peds.2020-026286. 33563769 10.1542/peds.2020-026286 PMC7919107

[39] Sonawane K, Zhu Y, Montealegre JR, Lairson DR, Bauer C, McGee LU, Giuliano AR, Deshmukh AA. Parental intent to initiate and complete the human papillomavirus vaccine series in the USA: a nationwide, cross-sectional survey. Lancet Public Health. 2020;5:e484–92. 10.1016/s2468-2667(20)30139-0. 32707126 10.1016/S2468-2667(20)30139-0 PMC7484349

[40] Glanz JM, Wagner NM, Narwaney KJ, Shoup JA, McClure DL, McCormick EV, et al. A mixed methods study of parental vaccine decision making and parent-provider trust. Acad Pediatr. 2013;13:481–8. 10.1016/j.acap.2013.05.030. 24011751 10.1016/j.acap.2013.05.030 PMC3767928

[41] Kowalik M. Ethics of vaccine refusal. J Med Ethics. 2022;48:240–3. 10.1136/medethics-2020-107026. 33637609 10.1136/medethics-2020-107026

[42] Wang Q, Yang L, Li L, Liu C, Jin H, Lin L. Willingness to vaccinate against herpes zoster and its associated factors across WHO regions: global systematic review and meta-analysis. JMIR Public Health Surveill. 2023;9:e43893. 10.2196/43893. 36892937 10.2196/43893 PMC10037179

[43] Zhang H, Chen L, Huang Z, Li D, Tao Q, Zhang F. The effects of parent’s health literacy and health beliefs on vaccine hesitancy. Vaccine. 2023;41:2120–6. 10.1016/j.vaccine.2023.02.026. 36822968 10.1016/j.vaccine.2023.02.026 PMC9943708

[44] Mamudu HM, Ahuja M, Adeniran E, Oke A, Hamilton B, Dowling-McClay K, Fletcher RA, Stewart DW, Collins J, Keener J, Paul TK, Weierbach FM. COVID-19 vaccine hesitancy and health literacy in US Southern States. Am J Manag Care. 2023;29:300–6. 10.37765/ajmc.2023.89371. 37341977 10.37765/ajmc.2023.89371

[45] Luisi MLR. From bad to worse: the representation of the HPV vaccine Facebook. Vaccine. 2020;38:4564–73. 10.1016/j.vaccine.2020.05.016. 32417141 10.1016/j.vaccine.2020.05.016

